# Structure–Property Behavior of Hydroxyl-Terminated Polybutadiene-Based Urethanes Additionally Cross-Linked Using Sustainable Biosourced Rosin Esters

**DOI:** 10.1021/acsapm.5c00220

**Published:** 2025-04-10

**Authors:** Aran Guner, Frank Lee, Daniel W. Lester, James S. Town, Steven Huband, Daniel Jubb, Ken Lewtas, Tony McNally

**Affiliations:** † International Institute of Nanocomposite Manufacturing (IINM), WMG, University of Warwick, Coventry CV4 7AL, U.K.; ‡ Polymer Research Technology Platform, University of Warwick, Coventry CV4 7AL, U.K.; § Department of Physics, University of Warwick, Coventry CV4 7AL, U.K.; ∥ The Falcon Project Limited, Manchester M29 7NW, U.K.

**Keywords:** hydroxyl-terminated polybutadiene (HTPB), rosin esters, miscibility, isocyanates, cross-linking, mechanical properties

## Abstract

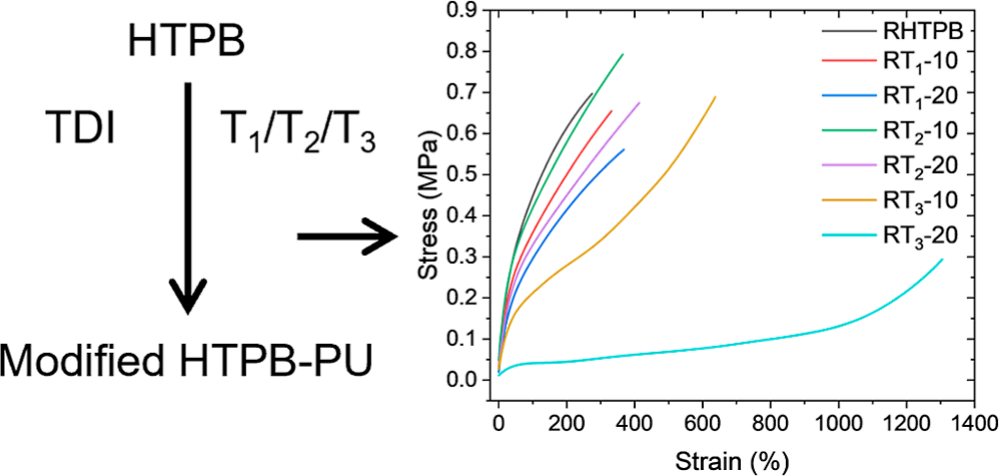

Hydroxyl-terminated polybutadiene (HTPB) particularly when cross-linked with a diisocyanate is a very versatile elastomer having excellent mechanical and low temperature properties suitable for applications as diverse as binders in rocket propellants to surface coatings. These properties can be tailored further by the inclusion of a plasticizer, e.g., octadecyl adipate, but there are many technical challenges remaining around the use of such plasticizers, including migration from and miscibility with HTPB, together with the problem that such plasticizers are synthesized from non-renewable feedstocks. To address these limitations, rosin and functional rosin esters, sourced from pine trees, were blended with HTPB at loadings up to 20 wt % prior to cross-linking with toluene diisocyanate. All rosin esters studied were shown to be fully miscible with HTPB; a single glass transition temperature (*T*_g_) was measured for all HTPB/rosin ester blends slightly above the *T*_g_ (−79 °C) of HTPB and well below that of the rosin esters (38–58 °C). Simultaneous wide-angle X-ray scattering (WAXS) and small-angle X-ray scattering (SAXS) measurements confirmed that there was no phase separation between the HTPB and rosin esters when blended. From increases in interdomain sizes, measured from X-ray scattering experiments on postcured samples, only the functional rosin ester (T_3_) takes part in the cross-linking reaction. Consequently, for the HTPB modified with T_3_ at 10 wt %, the elongation at break (ε) increased from 275% for unmodified HTPB to 600% and critically without a decrease in ultimate tensile strength (σ). For 20 wt % T_3_, ε increased to 1200%, and the material displayed strain-hardening behavior. The mechanical properties of HTPB can be tailored using functional rosin esters to alter the diisocyanate cross-linking reaction of the rubber.

## 1 Introduction

Hydroxyl-terminated polybutadiene (HTPB) is a liquid prepolymer commonly used as a binder in solid composite propellants when cross-linked with a diisocyanate to form a polyurethane (PU) network. It is used in a broad range of rocket motor formulations,^1−3^ coatings, and adhesives due to its mechanical and low temperature properties.^4−6^ Due to its widespread use, the properties of HTPB have been widely studied describing how HTPB microstructure,^7,8^ functionality,^6,8−11^ and molecular weight^6,8,9^ all impact the resultant mechanical and thermal properties of this elastomer. These factors are dependent on the HTPB synthesis method used as free radical, anionic, and ring opening metathesis polymerization yield different microstructures and properties.^12^ Free radical polymerization is most widely used for commercially produced HTPB via hydrogen peroxide-initiated polymerization of 1,3-butadiene. Despite the variation in mechanical properties due to the initial HTPB structure, research has focused on inclusion of additives that can alter the structure–property relationship of HTPB-based PUs. Current research has primarily focused on the use of plasticizers for processing and/or the type of isocyanate with the addition of diol and triol structures to alter the hard segment of the PU network.

Diisocyanates are used to form the hard cross-linking segment, and typically, toluene diisocyanate (TDI), methylene diphenyl diisocyanate (MDI), iso-phorone diisocyanate, and hexamethylene diisocyanate (HMDI) are commonly used, while the HTPB chains form the soft segment of the cross-linked PU. These structures not only determine the reactivity, but also the percentage of hard segment determines the mechanical properties of the PU rubber. Aromatic diisocyanates have been shown to have faster reaction rates than aliphatic diisocyanates, and MDI with two benzene rings has a faster reaction rate than TDI with only one benzene ring.^13^ The equivalence ratio, i.e., the ratio of isocyanate groups to hydroxyl groups, is used to tailor the mechanical properties within the range of 0.7–1.0, depending on the application of interest. Studies have shown that as the equivalence ratio increases, so does the tensile strength and modulus of the elastomer, while the elongation at break decreases.^10,14^ Additionally, the functionality of HTPB can alter the percentage of hard segment of the rubber, where the functionality describes the number of OH groups per HTPB chain and is typically around 2.5 for free-radically made commercial materials, but with an equal equivalence ratio, a HTPB with a higher functionality will cross-link to form a stiffer material.^9,10^

Diols and triols are introduced as additives to HTPB to provide further manipulation of the mechanical properties of the cross-linked system. Diols function as a chain extender of the HTPB and will result in an increase in elongation at break, while triols function as a cross-linker and result in a decrease in elongation at break but an increase in tensile strength.^14−16^ However, studies have shown that the inclusion of diols above a ratio of 2:1 for HTPB will result in increased tensile strength and reduced elongation at break.^3,16,17^ This behavior can be explained by the longer HTPB chains increasing the entanglement density to a point that the chains become stiffer with reduced mobility and flexibility.

A plasticizer can also be added as a processing aid to reduce the viscosity of the HTPB prepolymer, but its inclusion will also alter the structure–property relationship of the cross-linked system. The low temperature properties of HTPB can be critical depending on the application, and plasticizers can be added to lower the glass transition temperature (*T*_g_) of HTPB.^2^ Furthermore, its inclusion provides increased elasticity without negatively impacting tensile strength due to increased polymer chain mobility.^2,18^ The plasticizer does not chemically take part in the cross-linking reaction, rather it can migrate throughout the cross-linked network over time.^19−22^

Despite the success in tailoring the mechanical properties of HTPB–PUs, current research has shown that functional alcohols and plasticizers have limitations due to the negative correlation between tensile strength and elongation at break. Thus, it is common to see multiple additives being used in unison to achieve the required properties. In addition, limited research has been completed on organic based materials that could not only provide a more sustainable replacement to functional alcohols and plasticizers and surpass the current expectations of HTPB. Recently, we reported for the first time that rosin ester can participate in the cross-linking of HTPB with diisocyanates.^23^ Current uses for the bioderived rosin esters used in this study are as tackifiers for adhesive applications, in which their inclusion enhances the tack adhesion of the polymer substrate.^24^ Despite this class of additives having not been previously reported with HTPB, studies on tackifier inclusion in different polymer systems show promising benefits as a route to tailoring mechanical properties. The primary modification that tackifiers impart is a reduction in storage modulus, *G*′, a parameter used to determine the “flowability” and ability to wet a substrate which is vital for pressure-sensitive adhesives.^25−28^ In addition to a reduction in *G*′, an increase in elongation at break has also been reported, which could be attributed to the enhanced mobility of the polymer network as evidence from a reduction in *G*′.^25−27,29^

In this work, sustainable bioderived additives based on rosin esters and functional rosin esters were added to HTPB at loadings up to 20 wt %. The impact of rosin ester type and loading on HTPB–PU structure–property relationships is described, and how the resultant cross-linked structure obtained determines the enhanced HTPB mechanical properties reported.

## 2 Materials and Methods

### 2.1 Materials

Table 1 lists the materials used by name, code, and chemical structure in this study. For this study, all additives are coded T_*x*_, where *x* denotes the specific additive used relative to Table 1. T_*x*_-XX is used for all modified HTPB systems with XX detailing the weight percentage of the additive in that system. All cross-linked systems are prefixed with the letter R; as such, RT_1_-20 is cross-linked HTPB which contains additive one (see Table 1) at a loading of 20 wt %. The ^1^H NMR spectra of all additives and HTPB were recorded to confirm their structures, see the Supporting Information, Figures S1–S4. Figure S1 includes both the ^1^H NMR spectrum of HTPB and the calculations used to determine the microstructure and hydroxyl functional group distribution. Figures S2–S4 show the assignment of peaks for the ^1^H NMR spectra for T_1_, T_2_, where the functional hydroxyl groups are evident for T_3_. T_1_ does not contain an acid, but the acid numbers (mg KOH/g, ASTM D 465) for T_2_ and T_3_ are 5 and 15, respectively.

**Table 1:**
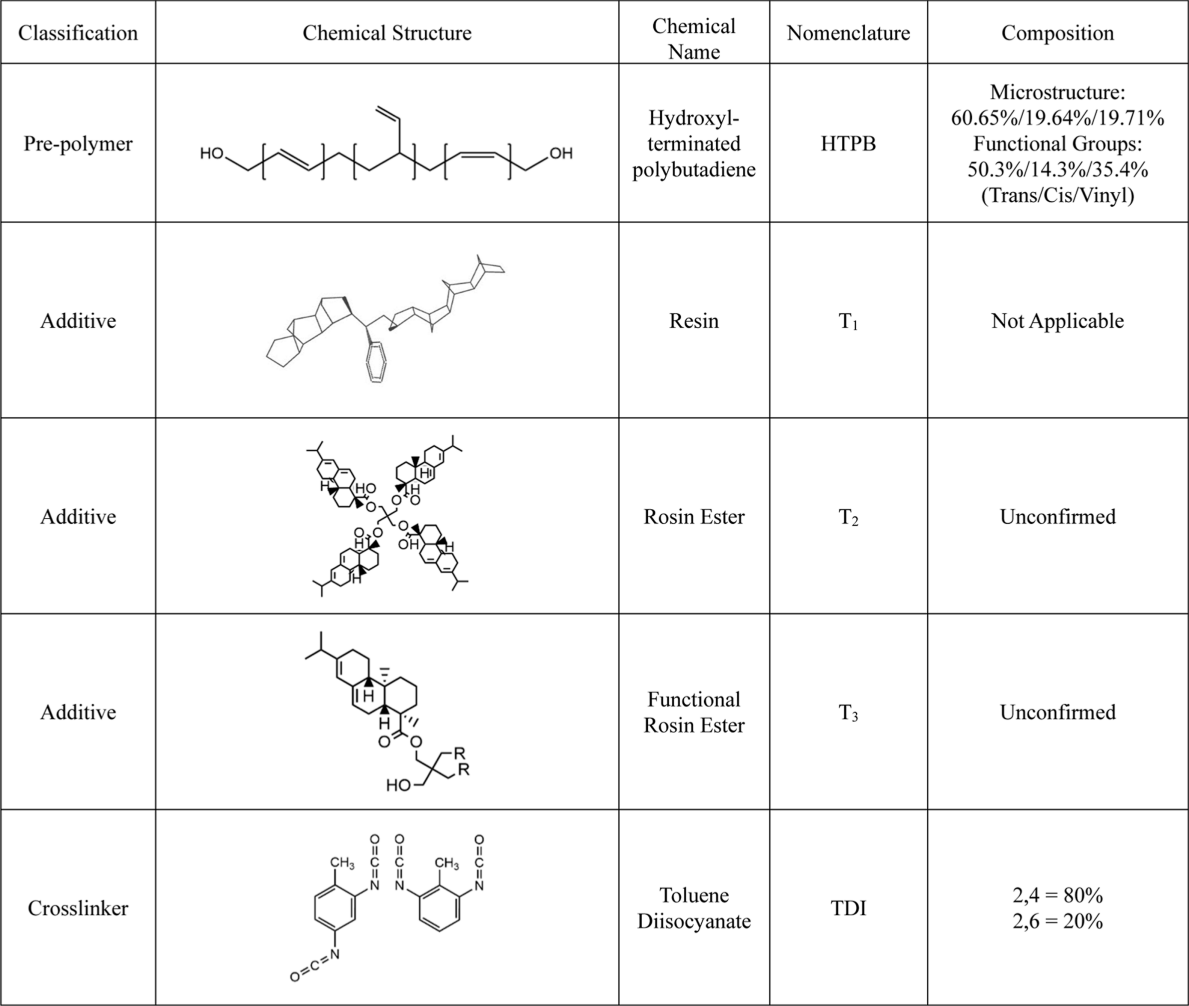
Materials Used during This Study

HTPB (*M*_w_ = 9803 g/mol, GPC, PS Standard, hydroxyl value = 0.75 mequiv/g) was supplied by the Falcon Project Ltd. and was stored under argon and dried before use. Prior to cross-linking, HTPB and the HTPB modified blends were dried under vacuum for 24 h at 60 °C. T_1_ (*M*_w_ = 690 g/mol, GPC, PS Standard), T_2_ (*M*_w_ = 1026 g/mol, GPC, PS Standard), and T_3_ (*M*_w_ = 1067 g/mol, GPC, PS Standard) were all supplied by the Falcon Project Ltd. and used as received. TDI (equivalent weight = 0.89 g/equiv) was supplied by Sigma-Aldrich and stored in a fridge at 4 °C prior to use.

### 2.2 Preparation of Modified HTPB and Cross-Linked Modified HTPB

**Scheme 1:**
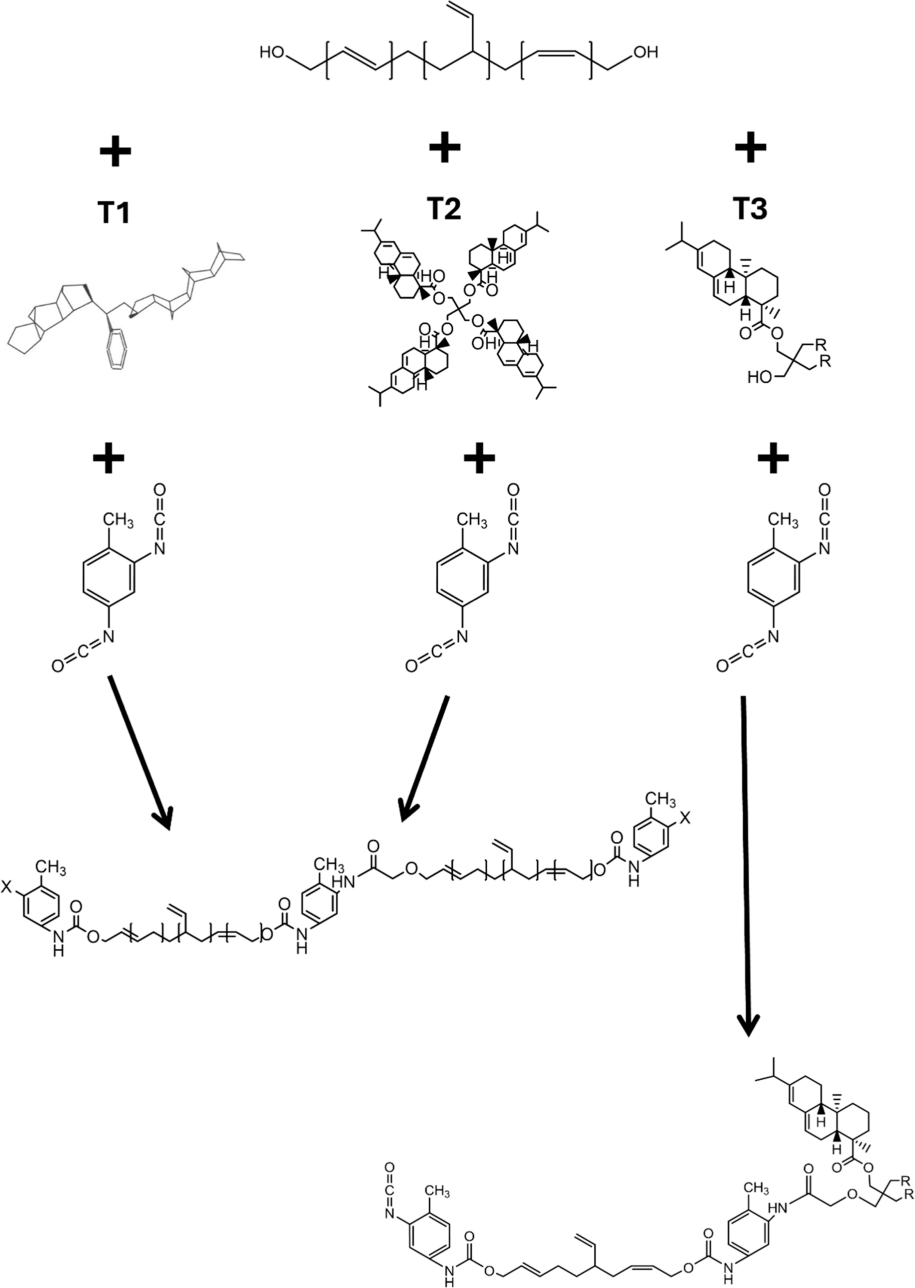
Cross-Linking Reactions of HTPB with TDI and T_1_, T_2_, and T_3_

Scheme 1 shows the reaction of the HTPB prepolymer with T_1_, T_2_, or T_3_. In all cases, the first step involves mixing HTPB and T_1_, T_2_, or T_3_ in a glass beaker under stirring for 15 min at 110 °C. 80 g of each modified HTPB was prepared for cross-linking, but only the weight of HTPB was used to determine the mass of curative required to achieve an equivalence ratio of 1.00. After addition of the TDI and 30 min mixing, the formulation was cast into a PTFE mold approximately 3 mm deep and placed in an oven at 60 °C for 5 days to cure.

### 2.3 Characterization

FTIR spectra were recorded in attenuated total reflectance (ATR) mode using a Bruker Tensor 27 Spectrometer fitted with a diamond ATR crystal. A resolution of 2 cm^–1^ and an average of 64 scans with a background scan were used to produce each spectrum in the range 500–4000 cm^–1^ and analyzed using OPUS software. FTIR spectroscopy was also used to track the HTPB cure reaction where scans were recorded using the same parameters as above every 2 min for the first 6 h and then every 30 min for up to 5 days, depending on the disappearance of the characteristic N=C=O peak at 2270 cm^–1^. The FTIR stage was maintained at 60 °C with a stainless steel centering ring containing the sample in the region of analysis. The percentage of the cure reaction completed was tracked using eq 1, where *C*_*XXXX*_ represents the area of that peak. The characteristic C=C band at 1640 cm^–1^ was used as a reference peak as this peak should remain unaltered throughout the reaction.^30^
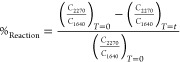
1

The thermal properties of the prepolymer blends were studied by Differential Scanning Calorimetry (DSC) using a TA Instruments DSC 2500 and evaluated using Origin software. Scans were completed using sample weights of 9.5 ± 4 mg in Tzero Aluminum Pin Hole Hermetic pans, first cooling to −120 °C (first cooling) at 10 °C/min, and holding for 5 min. Samples were then heated to 100 °C (first heating), held for 5 min, and subsequently cooled (second cooling) and heated (second heating) with 5 min isotherms between each heating and cooling step.

Simultaneous small-angle X-ray scattering (SAXS) and wide-angle X-ray scattering (WAXS) were used to study both the HTPB prepolymer and cross-linked HTPB systems. The prepolymer systems were studied using a Xenocs Xeuss 2.0 instrument with a wavelength of 1.54189 Å and an exposure time of 10 min. Hybrid photon counting detectors were used to capture WAXS (Pilatus 100K) images at a detector distance of 0.164(2) m and at two different distances to capture SAXS (Pilatus 300K) images at 0.338(3) and 2.481(5) m. The mean interdomain spacing (*d*) was calculated using *d* = 2π/*Q*_peak_, where *Q*_peak_ is the peak position in the SAXS data. The cross-linked systems were studied using the Diamond Light Source, Beamline I22. An X-ray wavelength of 1 Å (12.4 keV) was applied for an exposure time of 1 s. 2D SAXS and WAXS images were detected with a sample to detector distances of 9.7 and 0.18 m, respectively, on a Pilatus 2M detector and converted to 1D images using the DAWN software package.

A Triton Tritec 2000 DMTA instrument fitted with a standard air oven was used to obtain *E*′, *E*″, and tan δ as a function of temperature in the range of −105 to 100 °C. Tension geometry was selected due to the modulus and size of the samples. A free length of 10 mm was fixed between two tension clamps with a clamp mass of 9.34 g and a strain factor of 100 used. Samples with a width of 3.18 mm and a thickness of 3.1 ± 0.3 mm were tested. A pretension was applied to the sample to avoid buckling before the test chamber was cooled to −105 °C with liquid nitrogen and subsequently heated from −105 to 100 °C at a heating rate of 3 °C/min and a frequency of 1 Hz.

The chord modulus (MPa), ultimate tensile strength (UTS, MPa), and elongation at break (%) were determined from tensile mechanical testing using a Shimadzu Autograph AGS-X rig equipped with a 10 kN load cell. Extension was recorded with a twin TRViewX noncontact digital video extensometer using Trapezium X software. Specimens were cut to ASTM D 638—type V with a gauge length of 7.62 mm and an initial distance between grips of 25.4 mm. All samples were evaluated at room temperature and a constant crosshead speed of 50 mm/min. A minimum of five samples were evaluated to obtain an average and standard deviation. The chord modulus was calculated based on the slope of the chord in the strain range of 0% and 10%.

Solvent swelling tests were performed, and the Flory–Rehner equation was used to calculate the cross-link density. All results were obtained from an average by submersing three specimens, measuring 7 mm × 7 mm × 3 mm, in 100 mL of toluene for 48 h. The samples were then weighed, toluene was removed under vacuum over 2 h at 100 °C, and the samples were weighed again to calculate the cross-link density. This procedure is further detailed in ref (3) along with the necessary equations which have been reported in the Supporting Information, and the polymer–solvent interaction value used for HTPB–toluene was 0.36.

## 3 Results and Discussion

### 3.1 Prepolymer

**Figure 1:**
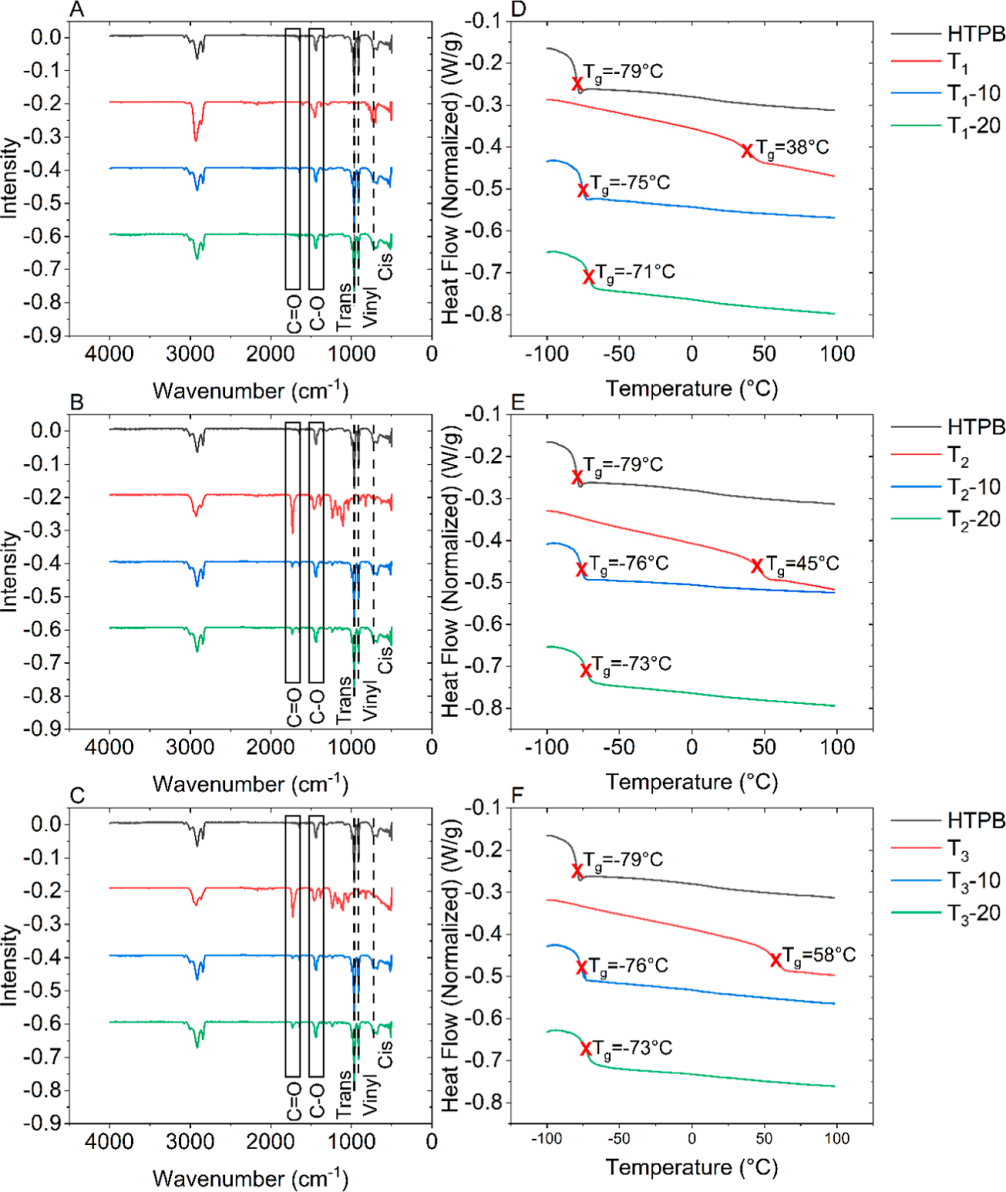
FTIR spectra for (A) T_1_-, (B) T_2_-, and (C) T_3_-modified HTPB and the corresponding DSC traces (D–F).

HTPB displays both cis- and trans-isomerism as well as vinyl double bonds, all of which determine the resultant properties of HTPB. The vinyl group is prone to aging, resulting in an increase in polymer stiffness as they are more likely to undergo cross-linking with neighboring double bonds. Due to the structure of trans double bonds, a highly trans polymer backbone is more likely to form a linear conformation providing high tensile strength but poor elongation at break; the opposite occurs with cis-structures which are more susceptible to coiling and allow for greater extensibility. Figure 1A–C shows the FTIR spectra of nonmodified and modified HTPBs with characteristic peaks at 724, 910, and 964 cm^–1^ for the cis, vinyl, and trans groups, respectively. These IR peaks remain unaltered for all modified formulations providing evidence that the modification of HTPB does not alter the elastomer backbone. In addition, no new peaks form, or existing peaks disappear for the modified HTPB formulations, as no reaction has taken place forming new bonds or altering the pre-existing bonding. Instead, IR peaks associated with the individual components are observed; this is more evident for T_2_- and T_3_-modified HTPB due to their chemical structure inducing peaks that are not obtained for HTPB. The most prominent of these is the C=O peak at approximately 1730 cm^–1^. As expected, when both T_2_ and T_3_ are incorporated into HTPB, the intensity of this peak significantly decreases due to the lower concentration of C=O present, but the peak is more intense for the 20 wt % formulations compared to 10 wt %.

Thermal properties are vital not only in understanding molecular transitions in a polymer network but also in understanding the miscibility of multiphase systems. For a two-phase polymer blend, a shift in the *T*_g_ of each polymer toward the other provides an insight into the degree of miscibility of the blend components. If all phases are fully miscible, then a multiphase system should go from having multiple *T*_g_’s to a single *T*_g_ depending on the percentage of each component present. Figure 1D–F shows the DSC traces for the second heating cycle for the T_1_, T_2_, and T_3_ systems, respectively. First, unmodified HTPB has a *T*_g_ at −79 °C, a property which can be favorable depending on application. In contrast, the additives all have *T*_g_’s above ambient temperature with the aromatic-modified aliphatic resin having the lowest at 38 °C and the functional rosin ester the highest at 58 °C. Despite this contrast in *T*_g_’s in all modified systems, the modified HTPB displays only a single *T*_g_ at a slightly elevated temperature from that of the unmodified HTPB at −79 °C. This indicates that all three rosins are fully miscible with HTPB as an immiscible system will show separate *T*_g_’s at the point of the pure system.^31^ This is not surprising as many resins/rosins were tested for miscibility (not reported here), and T_1_, T_2_, and T_3_ were chosen because of their good optical miscibility. However, despite T_1_ having the lowest *T*_g_, T_1_-10 and T_1_-20 have slightly higher *T*_g_’s compared to the corresponding T_2_ and T_3_ systems by 1 °C and increasing to 2 °C at 20 wt % are most probably within instrument error. A low *T*_g_ is associated with increased polymer chain flexibility and free volume between chains increasing mobility.^32^ Although the *T*_g_ increases with the introduction of all rosins, it can be inferred that T_1_ is the least effective in increasing the free volume and/or the flexibility of the polymer chain which results in the highest *T*_g_ despite having the lowest *T*_g_ itself. In addition, the same comparison can be made between T_2_ and T_3_. T_3_ has a *T*_g_ of 13 °C higher than T_2_, yet when introduced to HTPB has the same *T*_g_ at respective loadings (wt %), providing evidence that T_3_ provides an increase in free volume and/or flexibility of the HTPB chains compared to T_2_. This hypothesis can be confirmed from studying the cure behavior of and analysis of the physical properties of the cross-linked HTPB systems.

**Figure 2:**
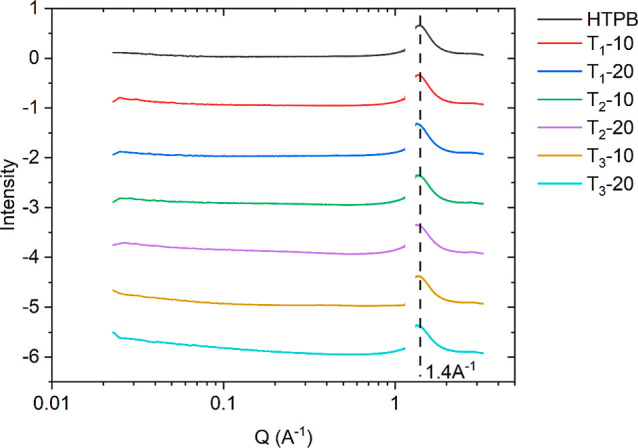
SAXS and WAXS plots of precured and modified HTPB.

Figure 2 provides both the SAXS and WAXS plots for precured HTPB and modified HTPB. The peaks in the WAXS region can indicate the crystallinity of a material, where crystalline structures appear as sharp peaks, while amorphous materials have a broad scattering peak as shown for HTPB with a peak position of 1.4 Å^–1^. The SAXS region provides detail on domain spacing which can occur due to ordering of different structures. In the precured material, no peaks are observed in this region. The lack of peaks in this region indicates that there is no phase separation between the additives and HTPB, in support of the observations made by DSC.

### 3.2 Cure Characteristics

**Figure 3:**
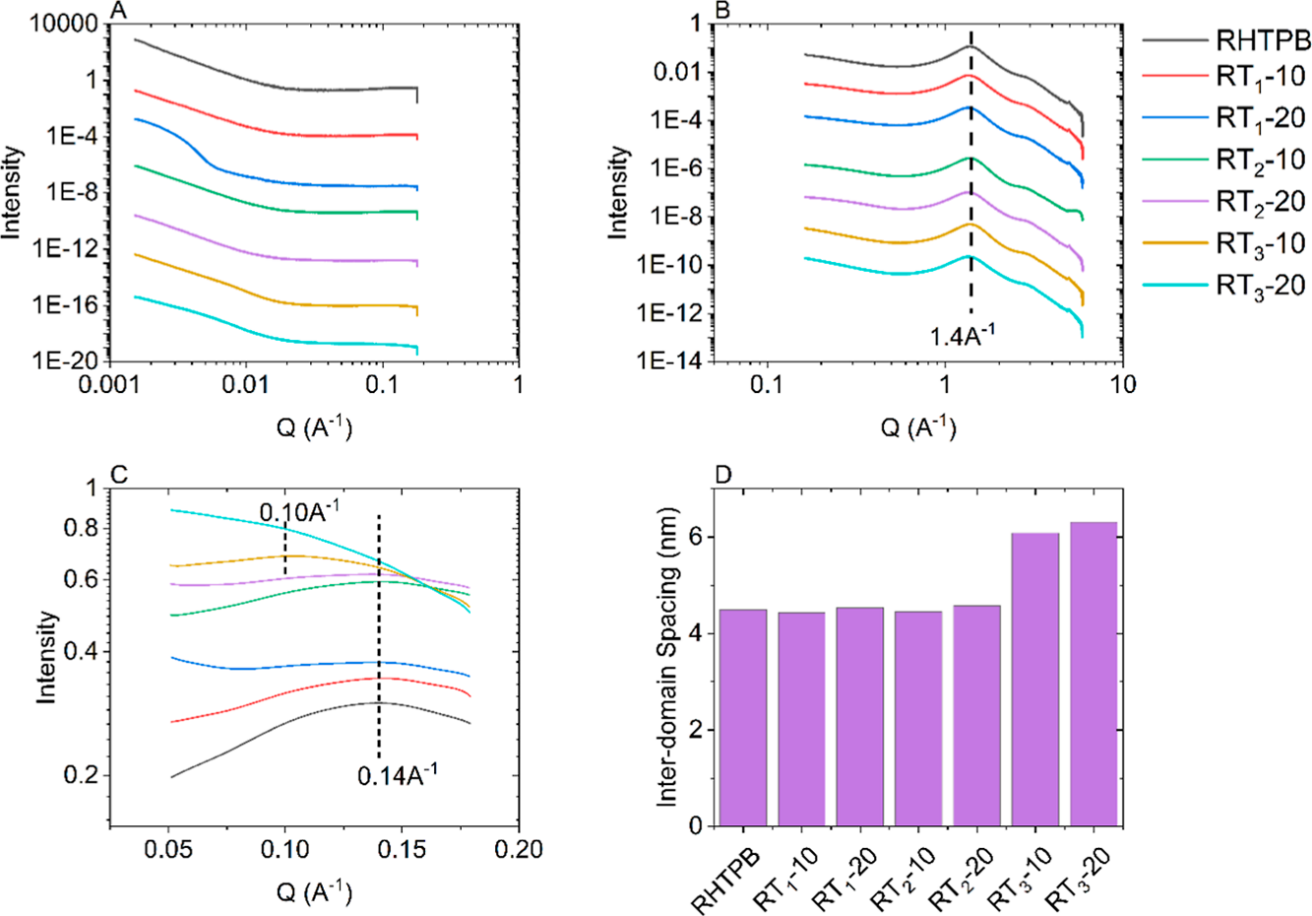
(A) SAXS and (B) WAXS plots of modified and unmodified cross-linked HTPBs and (C) zoomed view of the (A,D) corresponding plot of interdomain spacings.

X-ray scattering is a powerful technique to analyze the structure of materials, in particular crystalline polymers. However, the technique is not limited to crystalline materials and has been used previously to study amorphous HTPB-based PUs.^33^ Moreover, WAXS can provide an assessment of the intermolecular distances between macrochains. By comparison of the amorphous halo for both the prepolymer and cross-linked system, it is seen that the peak position is unaltered and so is the distance between macrochains (Figure 3B). This provides evidence that not only do the rosins not impact the HTPB backbone, as previously discussed, but neither does the cross-linking reaction. The cross-linking reaction does introduce the hard segment of the HTPB–PU, and segregation at the segmental level is due to the arrangement of a hard segment. Figure 3C (taken from Figure 3A) shows the peak in the SAXS region that is attributed to the interdomain spacing of the hard segment. Lucio^33^ has shown that this spacing can be altered due to the structure of the isocyanate used during the cross-link reaction with aromatic isocyanates having a smaller spacing than aliphatic isocyanates. As the same isocyanate, TDI, has been used in this work to form all PU networks, it was expected that all formulations would have the same interdomain spacing. This is true except for the case of RT_3_ formulations, in which the interdomain spacing increases from approximately 4–6 nm Figure 3D. This increase in interdomain size combined with the cure characteristics of T_3_ confirms that the functional hydroxyl groups of T_3_ take part in the cross-link reaction. That T_3_ has a bulky structure (see Scheme 1) and the increased interdomain spacing attributed to the hard segment of the PU would suggest a proportion of the T_3_ molecules react directly with the diisocyanate, thus “capping” the HTPB chain, instead of chain extending neighboring HTPB chains.

**Figure 4:**
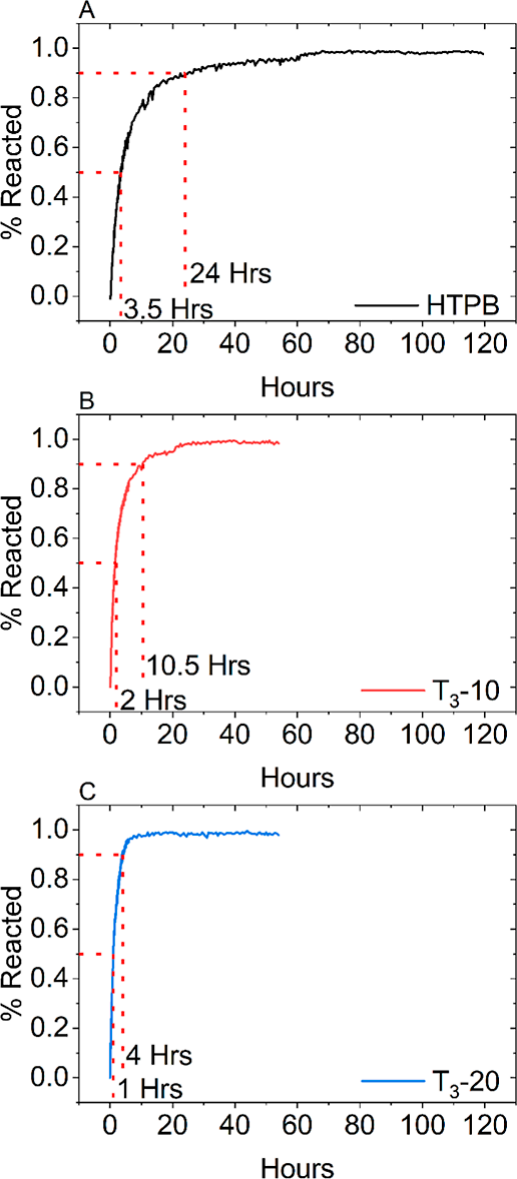
% NCO reacted as a function of time for (A) unmodified HTPB and functional rosin ester-modified HTPB at (B) 10 and (C) 20 wt %.

That addition of T_3_ altered the cross-linking of HTPB, shown from X-ray scattering experiments, and the disappearance of the NCO peak of TDI was recorded as a function of reaction time using FTIR. Figure 4A shows the time taken for the NCO to react with unmodified HTPB, and after approximately 3.5 h, that 50% of the NCO initially present has reacted with 90% reacted after 24 h. It is also noticeable that after ∼80% of the NCO has reacted, the rate of the reaction significantly starts to decrease. This is expected due to two factors, first the cross-linking reaction is completed for a 1:1 NCO/OH ratio. Second, the TDI used is an 80:20 w/w mixture of the 2,4- and 2,6-isomers. The 2,6-isomer has a greater steric hindrance due to the position of the CH_3_ group, which results in this isomer being less reactive; thus there is a slowing of rate of reaction in the latter part of the reaction by when the 2,4-isomer would have fully reacted.^13^Figure 4B,C shows the rate of reaction for T_3_-10 and T_3_-20, respectively. From these traces, the reaction rate is higher on introduction of T_3_ with 50% reacting after 2 h (T_3_-10) and 1 h (T_3_-20) and 90% reacting after approximately 10.5 h (T_3_-10) and 4 h (T_3_-20). In both systems, the NCO/OH was calculated to be 1:1 based on the weight percentage of HTPB. As T_3_ takes part in the cross-linking reaction, the true NCO/OH decreases with increasing T_3_ concentration and thus increases the probability of an NCO group interacting with an OH group. This also explains while there is a decrease in the rate of reaction in the latter stages due to the lower reactivity of the NCO 2,6-isomer, the difference in the rate of reaction is due to the greater abundance of OH groups. This behavior was further investigated by studying the cross-link density of the HTPB–PU systems.

### 3.3 Cross-Linked HTPB

**Figure 5:**
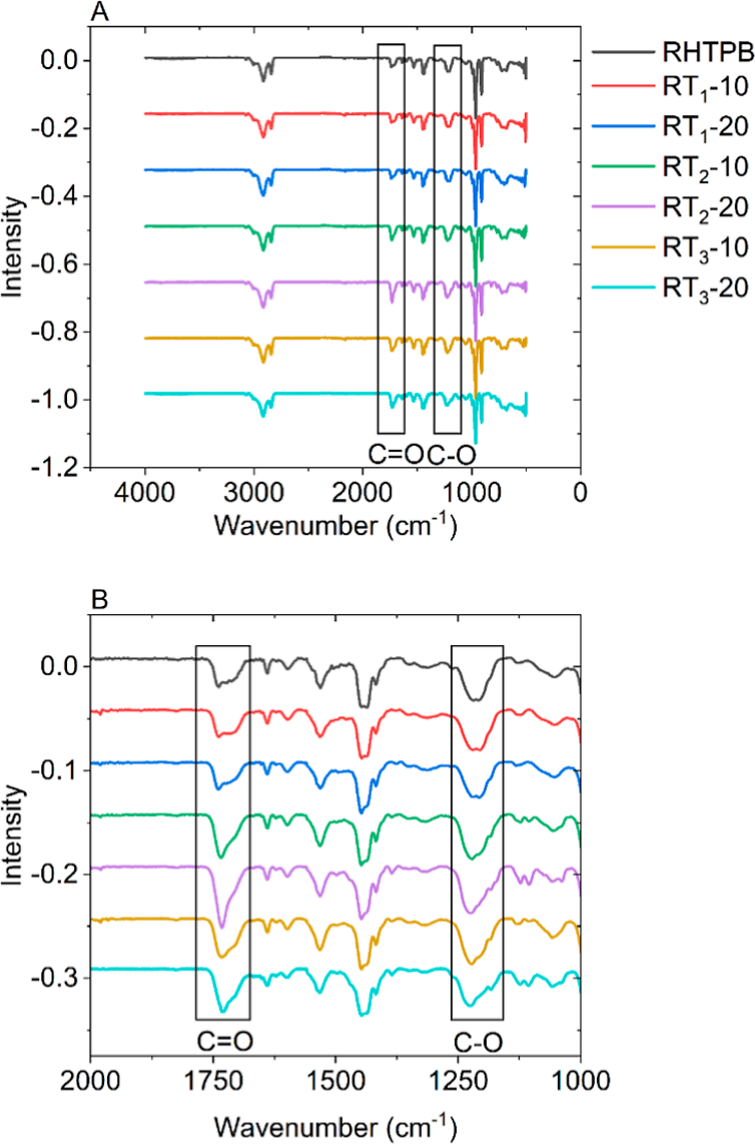
FTIR spectra of (A) cross-linked unmodified HTPB and modified HTPB and (B) a zoomed view in the region 2000–1000 cm^–1^.

The structure–property relationship of the cross-linked HTPB–PU systems was assessed by employing several techniques. Figure 5A,B shows the FTIR spectra of all cross-linked systems. With T_2_ and T_3_ both being rosin esters with the characteristic C=O peak, it is expected to see a change in peak intensity, as discussed above. However, it is also clear that there is a change in the shape of this peak as there can be contributions from different C=O bonding environments. The peak at 1740–1735 cm^–1^ is associated with C=O involved in negligible hydrogen bonding. In contrast, strong hydrogen bonding shifts this peak toward 1700 cm^–1^, and disorganized hydrogen bonding induces peaks around 1715 cm^–1^.^33^ When solely comparing the peak shapes from RT_2_-20 and RT_3_-20, in which peak changes are more readily observable, although all peaks are present, there is a shift toward an increase in hydrogen-bonded C=O peaks for RT_3_-20 with the peak position being approximately 1730 cm^–1^ compared to 1732 cm^–1^ for RT_2_-20 and 1739 cm^–1^ for RHTPB, respectively.

**Figure 6:**
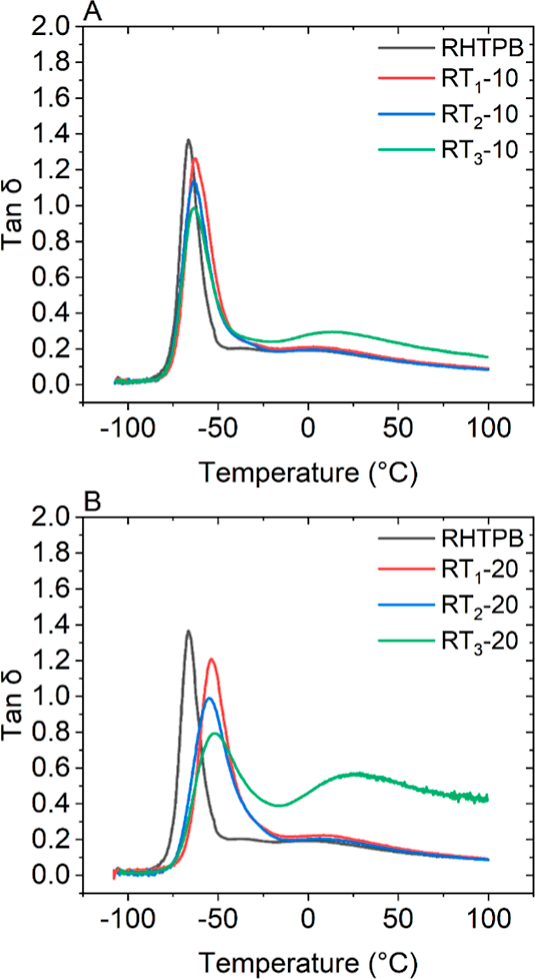
Plot of tan delta (δ) as a function of temperature for modified HTPB with (A) 10 and (B) 20 wt % T_1_, T_2_, and T_3_.

DMA was used to assess both the thermal transitions and understand the mobility of the polymer network postcuring. As HTPB is primarily used as an adhesive or as a binder, both tan δ and the storage modulus (*E*′/*G*′) are relevant properties, and determination of *T*_g_ from tan δ plots is critical. The shape of the peak also provides additional information on material elasticity and homogeneity. Figure 6A,B shows the tan δ curves of the cross-linked systems at 10 and 20 wt %, respectively, compared to the unmodified HTPB. There is a single *T*_g_ in all systems centered at −66 °C for RHTPB and rising to −63 °C for all modified HTPB with 10 wt % rosin. An increase in *T*_g_ is indicative of a decrease in mobility. This effect is magnified on addition of 20 wt % rosin to HTPB. However, there is a change in the *T*_g_’s at −54, −55, and −52 °C for RT_1_-20, RT_2_-20, and RT_3_-20, respectively. At the 20 wt % level, all additives are effective at restricting chain mobility with the functional rosin ester providing the greater hindrance, as expected. It can also be seen that RT_3_-10 and RT_3_-20 display a second transition, possibly associated with the relaxation of the PU hard segment present in all formulations. In RHTPB, RT_1_, and RT_2_ formulations, this relaxation is seen in the temperature range of −20 to 20 °C, but it increases for both T_3_ formulations to approximately 40 and 75 °C with the peak maxima at 14 and 25 °C for 10 and 20 wt %, respectively. This difference is due to the cure reaction and the resultant HTPB–PU structures, as seen in the SAXS data and an increase in hard segment size. Despite the increased restriction on polymer chain mobility, the decrease in the intensity of the tan δ peak is indicative of increased elasticity, and as such, the cross-linked HTPBs can be ranked RT_3_-20 > RT_3_-10 > RT_2_-20 > RT_2_-10 > RT_1_-20 > RT_1_-10 > RHTPB. This ranking should be reflected in the tensile mechanical properties of these materials. The width of the tan δ peak can show a degree of homogeneity in polymer structure; a slight increase is seen in all formulations at 10 wt %, which is expected to be due to the incorporation of the additive and increased with 20 wt % incorporation. RT_3_-20 shows a significantly wider peak, and this can be explained due to the increased volume of T_3_; it is anticipated to be a larger volume of T_3_-TDI hard segments and thus more variation with the expected HTPB-TDI hard segment.

HTPB is used in a variety of applications as they have relatively good mechanical properties which can be readily tailored by altering the cross-link density of this elastomer. One method employed to alter HTPB properties is by altering the NCO/OH equivalence ratio which impacts the ratio of hard to soft segment of the polymer network. However, increased hard segment will result in increased tensile strength but a reduction in the elongation at break (a measure of ductility) and vice versa.

**Figure 7:**
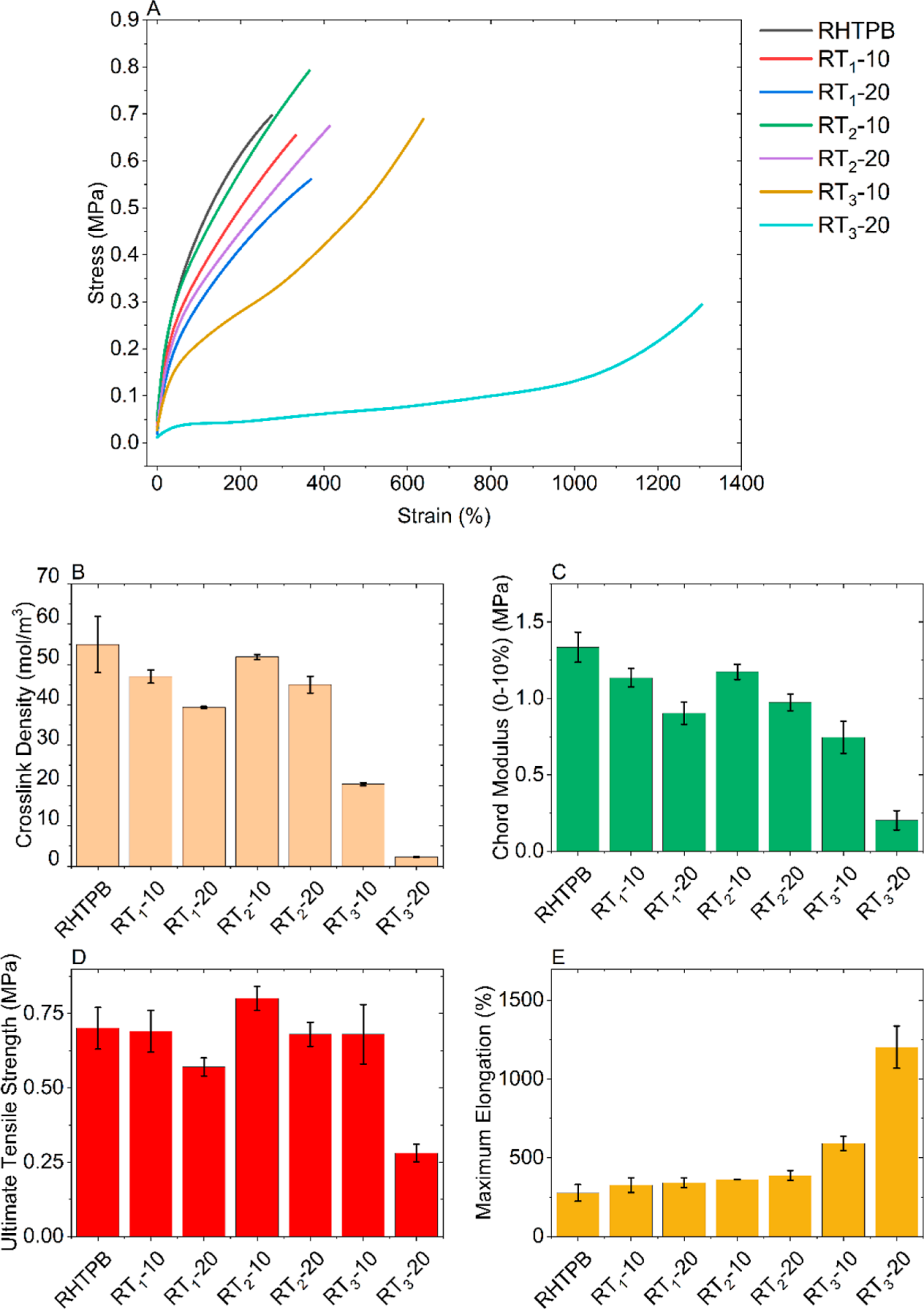
Change in (A) stress versus strain behavior, (B) cross-link density, (C) chord modulus, (D) UTS, and (E) maximum elongation for modified HTPBs.

Representative stress–strain curves are shown in Figure 7A, the cross-link density in Figure 7B, and the tensile mechanical properties of the modified HTPBs in Figure 7C–E. RHTPB has the highest cross-link density, as it is determined not only from the physical cross-links from the urethane bonding but also from chain entanglements. As discussed earlier, T_3_ takes part in the cross-linking reaction as it results in a decrease in the true NCO/OH, resulting in lower cross-link density. For T_1_ and T_2_, the cross-link density is reduced due to the chain entanglements. As already shown by DMTA, the flexibility of these chains is higher than for RHTPB, and thus it is expected there to be fewer chain entanglements which in turn explains the reduction in cross-link density for the higher concentration of T_1_ and T_2_. Despite this, the UTS values remain relatively consistent between RHTPB, RT_1_-10, RT_2_-10, and RT_2_-20 as it is primarily influenced by the physical cross-links, and with a consistent NCO/OH, there is little variation in UTS. Additionally, it is shown that T_2_ formulations have a higher UTS than T_3_ formulations at the corresponding rosin loading, with RT_2_-10 even surpassing RHTPB. The increase in tensile strength can be explained by the polarity of the rosin ester promoting a greater degree of intermolecular forces (interfacial interaction) compared to RHTPB and the RT_1_ formulations.

However, this does not explain why RT_3_-10 also exhibits an UTS matching RHTPB. In Figure 7A, there is a key difference in the stress strain plots observed for the T_3_ formulations; the phenomenon of strain hardening is seen in which an increase in stress is obtained at higher strains. This can occur in polymers due to the alignment of polymer chains at higher strains increasing the intermolecular forces between chains. As this behavior was not obtained for the other formulations, it is expected that the presence of the T_3_ molecule at some critical concentration at cross-links sites is responsible for inducing strain hardening behavior. Similar to that for T_2_, the polarity of the rosin ester when the polymer chains are aligned could cause a significant increase in the forces needed to overcome them, especially when in addition, the true NCO/OH is lower, resulting in a greater abundance of OH groups present.

This reduction in true NCO/OH is also partially responsible for the significant increase in the elongation at break, behavior that has been studied since the 1990s.^10^ Also, there is correlation between the height of the tan δ peak (elasticity) with the maximum elongation at break with RHTPB having the lowest elongation at about 275%, but this increases significantly to over 600% for RT_3_-10 but to 1200% for RT_3_-20.

## 4 Conclusions

The modification of HTPB with rosin and functional rosin esters was readily achieved. These additives are miscible with HTPB with a minimal increase in *T*_g_, a fundamental property for many applications, including for adhesives and as a binder in composite rocket propellants. The functional rosin ester (T_3_) participates in the cross-linking reaction and is part of the cross-linked network. This not only alters the cross-linking behavior but provides significant advantage in that the maximum elongation increased up to 600% at the 10 wt % level compared to 275% (for unmodified HTPB) but with a minimal reduction in tensile strength. For 20 wt % T_3_, the maximum elongation increased to 1200% but the decrease in tensile strength was ∼60% compared to RHTPB. T_1_ and T_2_ do not take part in the cross-linking reaction but show a similar trend in tensile mechanical properties with increasing maximum elongation with minimal impact on tensile strength. Unlike commonly used additives, such as functional alcohols and plasticizers, inclusion of these classes of rosin additives to HTPB can modify the maximum elongation of the elastomer without the deterioration of tensile strength with the additional benefit of being a sustainably sourced material suitable for a wide range of applications.

## Supporting Information Available

NMR spectra for the materials used in the study with the equations used to determine HTPB microstructure and functional group distribution and the corresponding equations used to determine HTPB cross-link density from solvent swelling experiments (PDF)
