# Supplementary material for: Structure–Property Behavior of Hydroxyl-Terminated Polybutadiene-Based Urethanes Additionally Cross-Linked Using Sustainable Biosourced Rosin Esters
Source: ACS Appl Polym Mater. 2025 Apr 10;7(8):4963–72. doi: 10.1021/acsapm.5c00220 (PMC12038791; doi:10.1021/acsapm.5c00220)
Supplement: Supplementary file 1 — ap5c00220_si_001.pdf [file ap5c00220_si_001.pdf]

Supporting Information

Structure-Property Behaviour of Hydroxyl-Terminated Polybutadiene based Urethanes Additionally Crosslinked using Sustainable Bio-sourced Rosin Esters

Aran Guner[1], Frank Lee[1], Daniel W. Lester[2], James S. Town[2], Steven Huband[3], Daniel Jubb[4], Ken Lewtas [1,4], Tony McNally[1]*

[1]International Institute of Nanocomposites (IINM), WMG, University of Warwick, Coventry, CV4 7AL, UK

[2]Polymer Research Technology Platform, University of Warwick, CV4 7AL and

[3]Department of Physics, University of Warwick, Coventry, CV4 7AL, UK

[4]The Falcon Project Limited, Manchester, M29 7NW, UK

Corresponding Author: Tony McNally, e-mail: t.mcnally@warwick.ac.uk

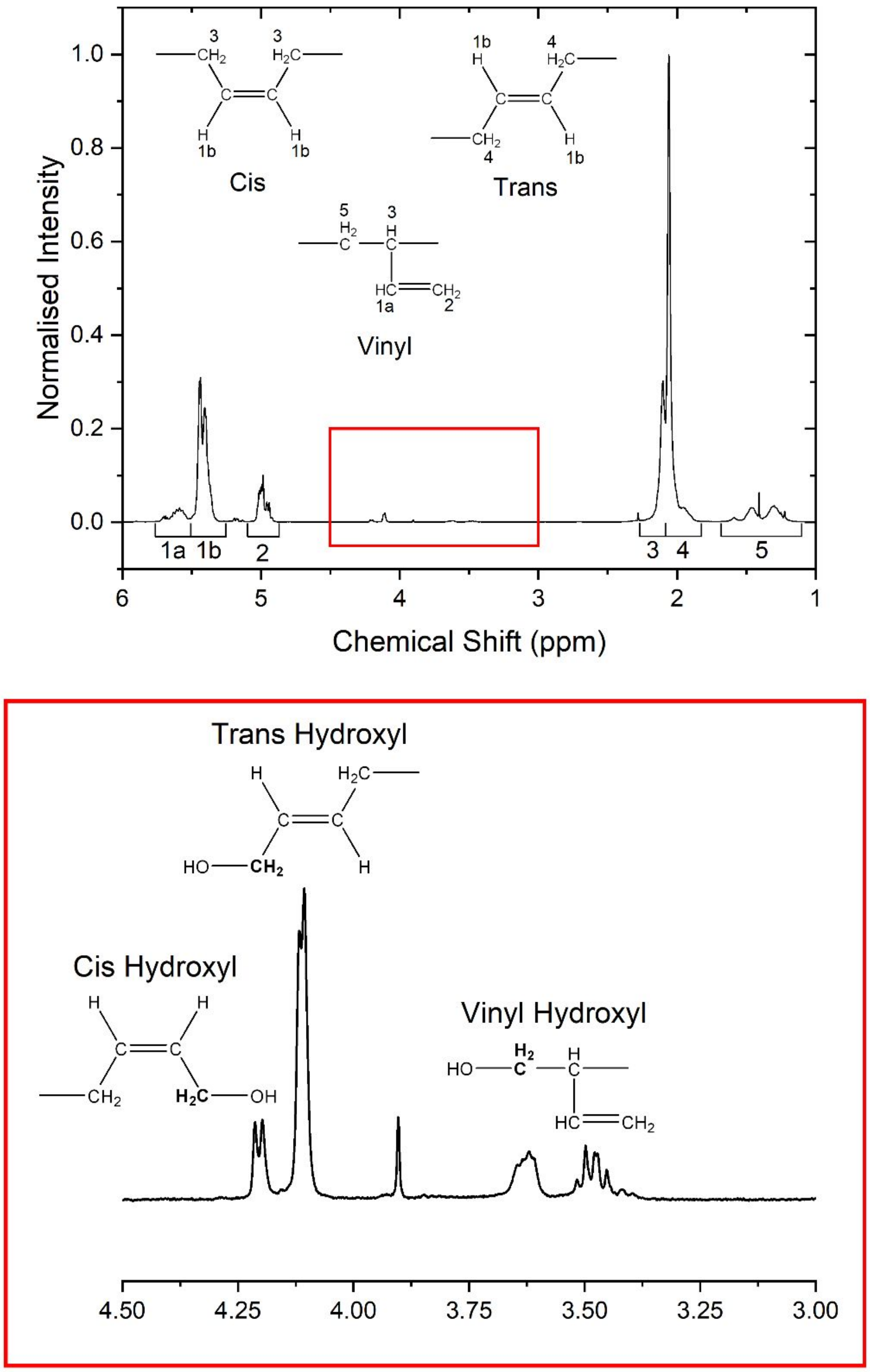

Figure S1. ^1H NMR spectrum of a) HTPB and b) enlarged view between 3ppm and 4.5ppm showing hydroxyl functional groups.

The equations used to calculate the microstructure and functional distribution are shown below where A_X denotes the area of the peak for the corresponding structure.

$$\% \, Cis = \frac{(2A_3 - A_2)(2A_1 - A_2)}{(2A_3 - A_2 + 2A_4)(2A_1 + A_2)} \quad (1)$$

$$\% \, Trans = \frac{2A_4(2A_1 - A_2)}{(2A_3 - A_2 + 2A_4)(2A_1 + A_2)} (2)$$

$$\% \, Vinyl = \frac{2A_2}{(2A_1 + A_2)} (3)$$

$$\% \, Cis(OH) = \frac{A_{cis(OH)}}{\left(A_{cis(OH)} + A_{Trans(OH)} + A_{Vinyl(OH)}\right)} (4)$$

$$\% \, Trans(OH) = \frac{A_{Trans(OH)}}{\left(A_{cis(OH)} + A_{Trans(OH)} + A_{Vinyl(OH)}\right)} (5)$$

$$\% \, Vinyl(OH) = \frac{A_{Vinyl(OH)}}{\left(A_{cis(OH)} + A_{Trans(OH)} + A_{Vinyl(OH)}\right)} (6)$$

T_1

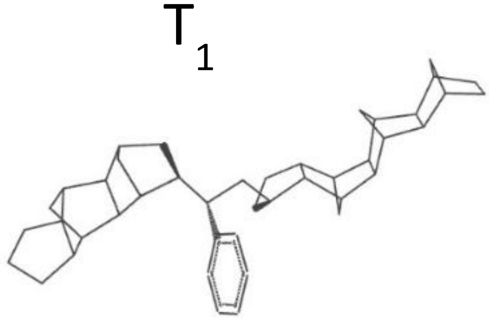

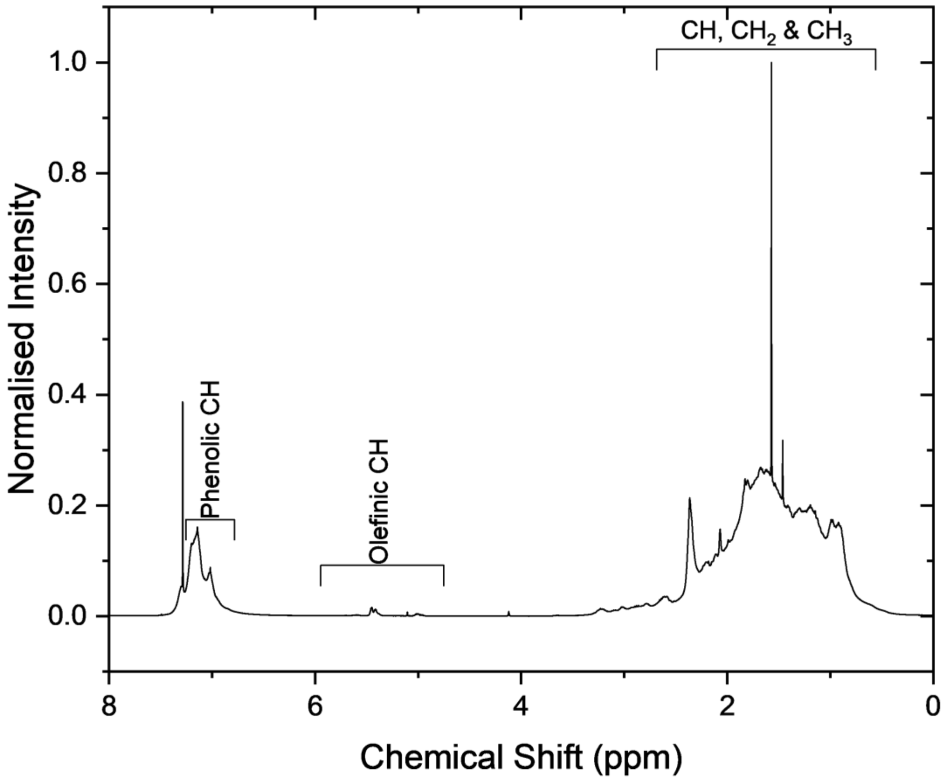

Figure S2.^{1}H NMR spectrum of Resin (T_1)

T2

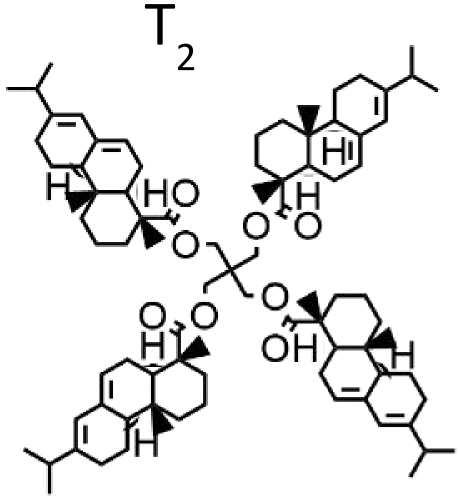

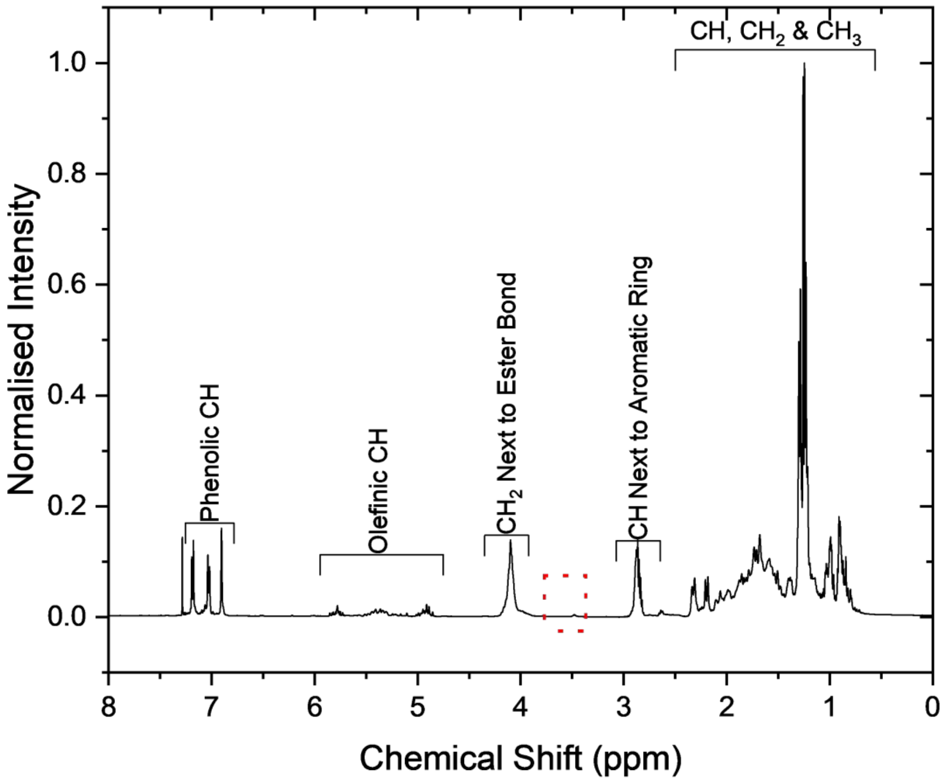

Figure S3. ^{1}H NMR spectrum of Rosin Ester (T_2)

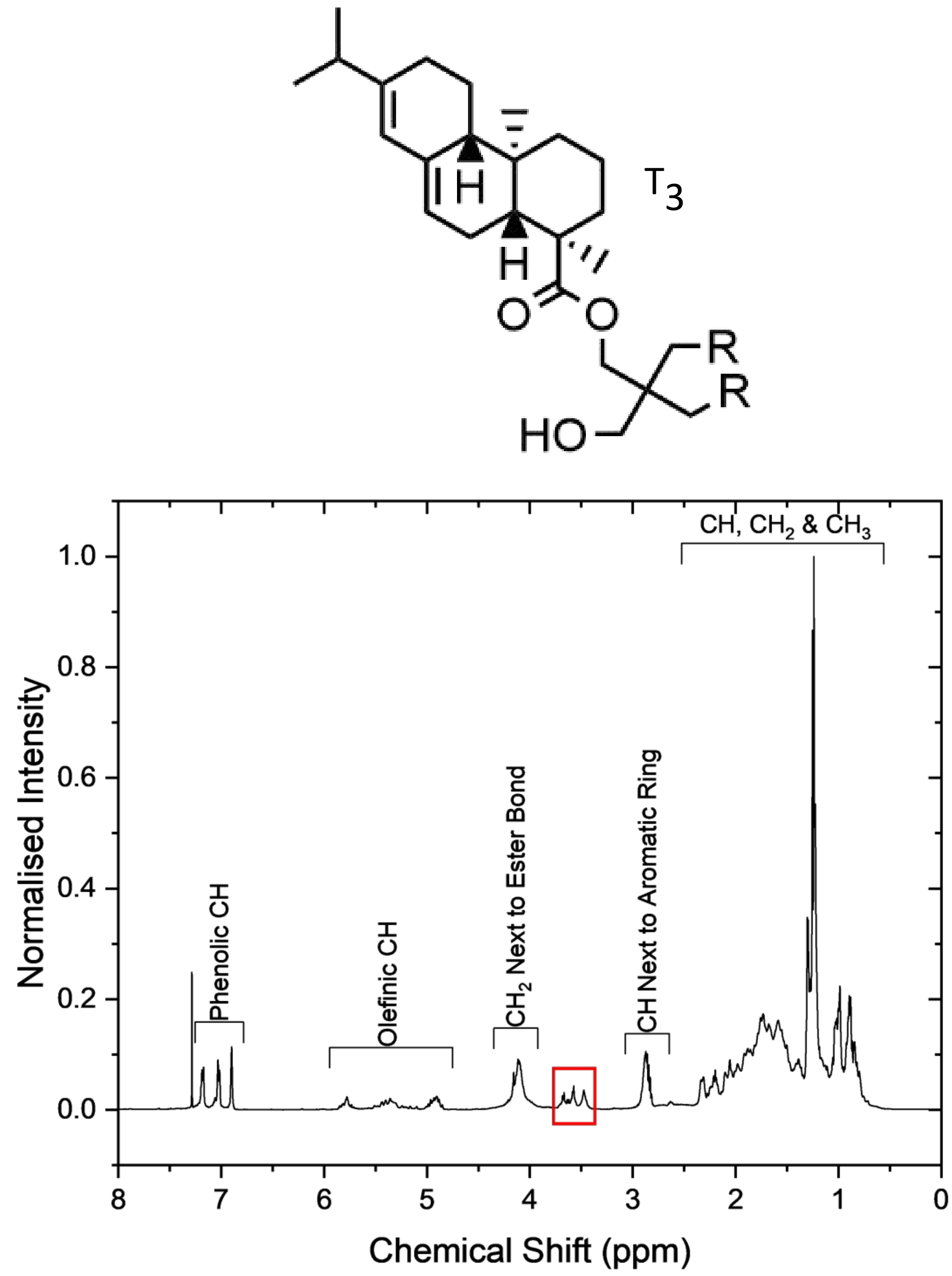

Figure S4. ^{1}H NMR spectrum of Functional Rosin Ester (T_3)

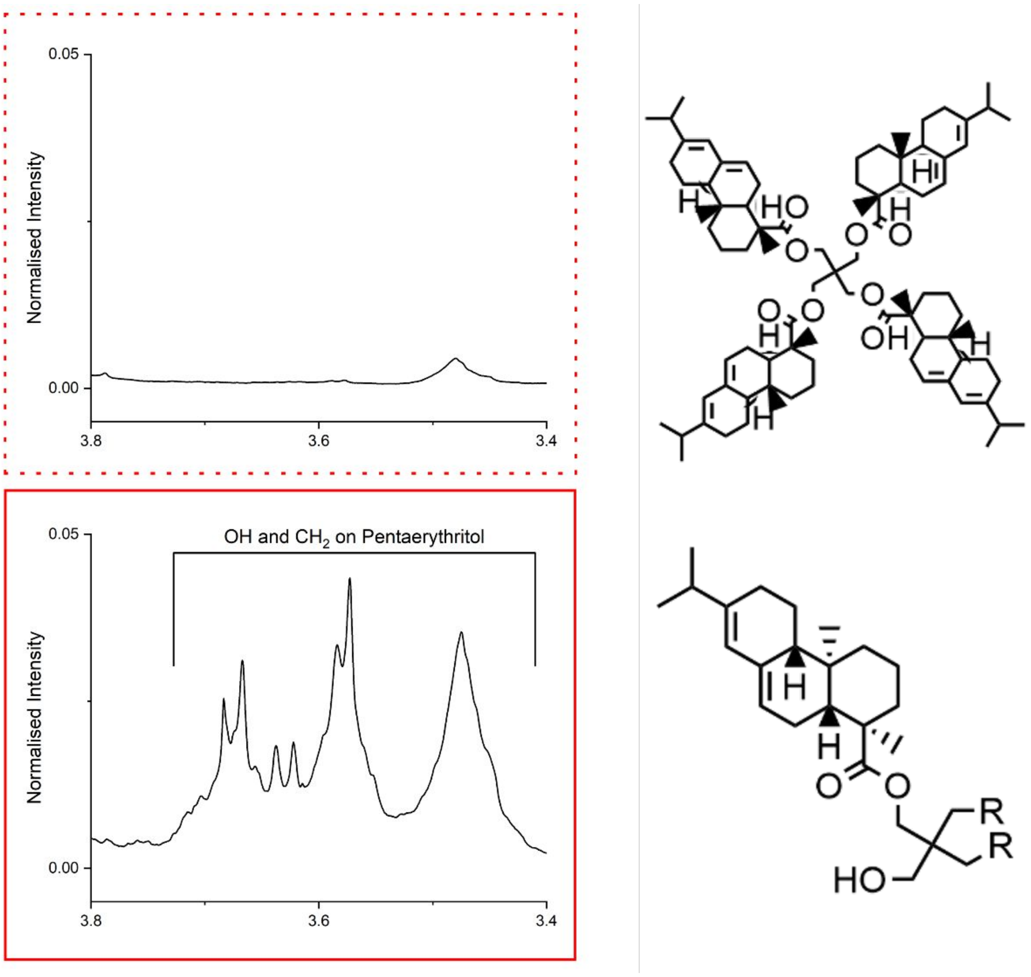

T_2

T_3

Figure S5. Comparison of the ^{1}H NMR spectra for T_2 and T_3 rosin esters between 3.4ppm and 3.8ppm to show the difference in hydroxyl functional groups.

The above ^{1}H NMR spectra show differences in chemical shift in the region 3.4 to 3.8ppm. The small peak at ~3.44ppm seen for T_2 is commonly found in rosin esters reacted with pentaerythritol and paired with a higher chemical shift of ~4.1ppm, see Figures S3 and S4. However, for T_3 the peaks are more intense as they also share the chemical shift of CH_2 adjacent to an OH group with the OH group having the higher chemical shift of ~3.65ppm [1].

Procedure for determining the crosslinking density.

The calculations followed to determine the crosslink density of the HTPB can be found in [2], in which the polymer-solvent interaction parameter is quoted as 0.36 for the case of HTPB-toluene.

$$Q = \left({}^{w_s}/_{w_{ds}}\right) - 1 \quad (7)$$

where, Q is the swelling ratio, and w_s and w_{ds} are the swollen and de-swollen weights of the sample, respectively.

$$w_2 = \frac{1}{(1+Q)} \ and \ w_1 = 1 - w_2 \quad (8)$$

where, w_2 and w_1 are the weight fractions of the polymer and the solvent, respectively.

$$v_2 = \frac{\left({}^{w_2}/_{d_2}\right)}{\left(\left({}^{w_1}/_{d_1}\right) + \left({}^{w_2}/_{d_2}\right)\right)} \quad (9)$$

where, v_2 is the volume fraction of the polymer and d_1 and d_2 are the densities of the solvent and polymer, respectively.

$$v_e = \frac{-\left(\ln(1 - v_2) + v_2 + \chi v_2{}^2\right)}{V_s\left(v_2^{\frac{1}{3}} - {}^{v_2}/_2\right)} \quad (10)$$

where, v_e is the crosslink density, V_s is the molar volume of the solvent and χ is the polymer-solvent interaction value.

(1) H4R Consortium. *Reference Document on the Identification of Rosin and Rosin Derivatives*; Penman Consulting: Brussels, Dec. 2012. https://h4rconsortium.com/attachments/Reference%20Document%20Rosin%20Derivatives%20V2%20January%202012.pdf (accessed 2021-08-12)

(2) Sekkar, V.; Alex, A. S.; Kumar, V.; Bandyopadhyay, G. G. Theoretical Evaluation of Crosslink Density of Chain Extended Polyurethane Networks Based on Hydroxyl Terminated Polybutadiene and Butanediol and Comparison with Experimental Data. *Journal of Energetic Materials.* **2018**, *36* (1), 38–47. DOI: 10.1080/07370652.2017.1307884.
